# Elevated intracellular cyclic‐di‐GMP level in *Shewanella oneidensis* increases expression of *c*‐type cytochromes

**DOI:** 10.1111/1751-7915.13636

**Published:** 2020-07-30

**Authors:** Chun Kiat Ng, Jiabao Xu, Zhao Cai, Liang Yang, Ian P. Thompson, Wei E. Huang, Bin Cao

**Affiliations:** ^1^ Department of Engineering Science University of Oxford Parks Road Oxford OX1 3PJ UK; ^2^ Singapore Centre for Environmental Life Sciences Engineering Nanyang Technological University Singapore City Singapore; ^3^ School of Civil and Environmental Engineering Nanyang Technological University Singapore City Singapore; ^4^ Present address: School of Medicine Southern University of Science and Technology Shenzhen China

## Abstract

Electrochemically active biofilms are capable of exchanging electrons with solid electron acceptors and have many energy and environmental applications such as bioelectricity generation and environmental remediation. The performance of electrochemically active biofilms is usually dependent on *c*‐type cytochromes, while biofilm development is controlled by a signal cascade mediated by the intracellular secondary messenger bis‐(3ʹ‐5ʹ) cyclic dimeric guanosine monophosphate (c‐di‐GMP). However, it is unclear whether there are any links between the c‐di‐GMP regulatory system and the expression of *c*‐type cytochromes. In this study, we constructed a *S. oneidensis* MR‐1 strain with a higher cytoplasmic c‐di‐GMP level by constitutively expressing a c‐di‐GMP synthase and it exhibited expected c‐di‐GMP‐influenced traits, such as lowered motility and increased biofilm formation. Compared to MR‐1 wild‐type strain, the high c‐di‐GMP strain had a higher Fe(III) reduction rate (21.58 vs 11.88 pM of Fe(III)/h cell) and greater expression of genes that code for the proteins involved in the Mtr pathway, including CymA, MtrA, MtrB, MtrC and OmcA. Furthermore, single‐cell Raman microspectroscopy (SCRM) revealed a great increase of *c*‐type cytochromes in the high c‐di‐GMP strain as compared to MR‐1 wild‐type strain. Our results reveal for the first time that the c‐di‐GMP regulation system indirectly or directly positively regulates the expression of cytochromes involved in the extracellular electron transport (EET) in *S. oneidensis*, which would help to understand the regulatory mechanism of c‐di‐GMP on electricity production in bacteria.

## Graphical abstract

*Shewanella* *oneidensis* MR‐1 strain with a higher cytoplasmic c‐di‐GMP level had a higher Fe(III) reduction rate and greater expression of genes that code for the proteins involved in the Mtr pathway. Single‐cell Raman microspectroscopy revealed a great increase of c‐type cytochromes in the high c‐di‐GMP strain as compared to MR‐1 wild‐type strain. c‐di‐GMP regulation system indirectly or directly positively regulates the expression of cytochromes involved in the extracellular electron transport (EET) in *S. oneidensis*.

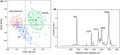

## Introduction

Bacteria can switch between planktonic and biofilm modes of growth (Hall‐Stoodley *et al*., 2004). Unlike their free‐swimming planktonic counterparts, bacteria in biofilms colonise surfaces and interfaces by anchoring themselves with self‐produced extracellular polymeric substances such as carbohydrates, proteins and nucleic acids, eventually forming a complex three‐dimensional matrix that encases the cells (Branda *et al*., 2005; Cao *et al*., 2011). Compared with the planktonic mode of life, the biofilm mode confers several key potential advantages for the bacteria which include, but not limited to, protection against predation, increased resistance to antimicrobial agents, facilitated genetic exchange and mutation to increase genetic variation in the gene pool (Flemming and Wingender, 2010; Flemming, 2011; Flemming *et al*., 2016). As such, the biofilm mode of life is the predominant form of existence for bacteria in almost all natural environments where they enable their communities to strive even in extreme and hostile conditions (Flemming and Wuertz, 2019). Such resilience and robustness exhibited by biofilms increases opportunities for their exploitation in environmental and industrial applications such as bioremediation, biocatalysis and bioelectricity generation (Singh *et al*., 2006; Gross *et al*., 2007; Tsoligkas *et al*., 2011; Zhang *et al*., 2014; Ng *et al*., 2017).

Many potential environmental and industrial applications of biofilms centre around the ability of electroactive bacteria (electrogen), for example *Geobacter sulfurreducens* and *Shewanella oneidensis*, to perform extracellular electron transfer (EET) and exhibit reductive and oxidative (redox) reactions, such as bioelectricity generation via microbial fuel cell and the immobilization of uranium using biofilms (Rosche *et al*., 2009; Cao *et al*., 2010; Halan *et al*., 2010; Yates *et al*., 2014; Liu *et al*., 2015). Electrogens can transfer electrons to exogenous insoluble acceptors mainly by three EET mechanisms: direct electron transfer, indirect electron transfer via shuttling of excreted mediators (e.g. flavins), and through electrically conductive nanowire or pili (Reguera *et al*., 2006; Von Canstein *et al*., 2008; Okamoto et al., 2011, 2012, 2014; Pirbadian *et al*., 2014). Ideally, bacteria should be physically close to the insoluble electron acceptor by forming a biofilm on its surface for energy‐efficient direct electron transfer to occur via *c*‐type cytochromes on the outer surface of the cell membrane. Hence, there may be possible links between the regulatory system of biofilm formation and cytochrome expression in these bacteria.

Control mechanism of bacterial biofilm formation and dispersal is closely associated with the signal cascade mediated by the intracellular secondary messenger bis‐(3ʹ‐5ʹ) cyclic dimeric guanosine monophosphate (c‐di‐GMP; Hengge, 2009). Intracellular regulation of c‐di‐GMP in bacteria cells is achieved through production by diguanylate cyclases and degradation by phosphodiesterases (Ryjenkov *et al*., 2005; Schmidt *et al*., 2005; Ryan *et al*., 2006) and regulated by extracellular environmental cues (e.g. nutrient availability and quorum sensing) detected by multiple sensory systems on and within the cells. C‐di‐GMP has been reported to influence the switching between the attached biofilm and motile planktonic mode of life in many microbes, stimulating the secretion of extracellular polymeric substances and affecting cell motility and virulent capabilities (Gjermansen *et al*., 2006; Jenal and Malone, 2006; Wolfe and Visick, 2008; Broberg *et al*., 2011; Spurbeck *et al*., 2012). Although the c‐di‐GMP regulatory system and EET pathway are involved in different aspects of biofilm attachment, the c‐di‐GMP regulatory system on electricity production in bacteria is still unclear.

In this study, we constructed a *S. oneidensis* MR‐1 mutant with a higher concentration of intracellular c‐di‐GMP and compared its transcriptomic and functional data with the MR‐1 wild‐type (WT), to examine the effect of the elevated c‐di‐GMP level on *c*‐type cytochrome expression. We further estimated *c*‐type cytochromes in *S. oneidensis* MR‐1 at the single‐cell level by employing single‐cell Raman microspectroscopy (SCRM; Xu *et al*., 2017). The results provide new insights into the effect of an elevated intracellular c‐di‐GMP concentration on the expression of cytochromes and open up new opportunities in the regulation of EET towards reproducible and controllable biofilm processes in environmental and industrial applications.

## Results and discussion

### Elevated intracellular c‐di‐GMP level enhanced S. oneidensis biofilm formation

To investigate whether c‐di‐GMP influenced the expression of *c*‐type cytochromes, we first constructed a *S. oneidensis* strain that constitutively expressed YedQ, a *Escherichia coli* diguanylate cyclase that catalyses the synthesis of c‐di‐GMP, which had been cloned into pBBR1MCS‐5 (MR‐1/pYedQ_2_).

**Fig. 1:**
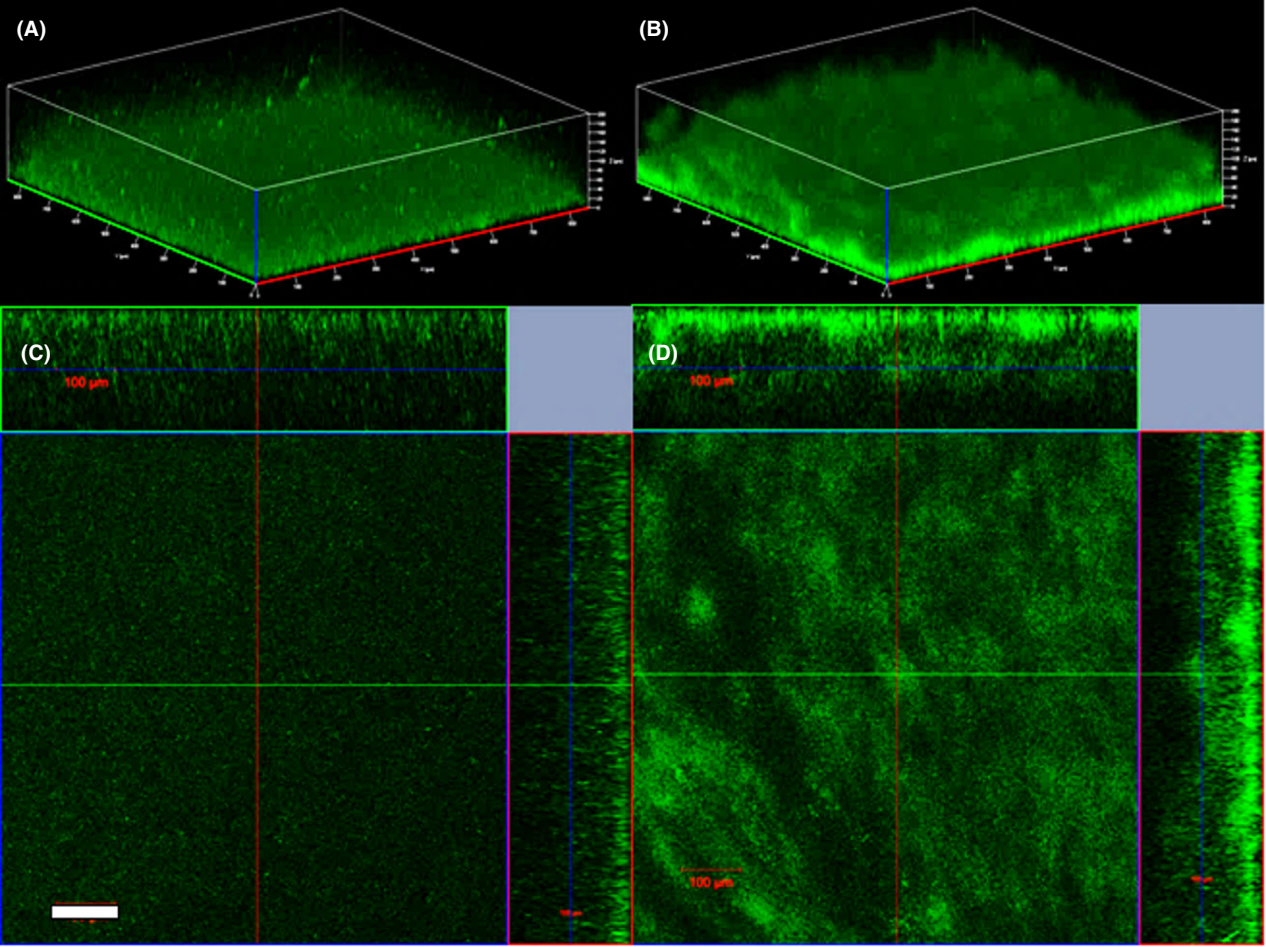
Representative confocal Laser Scanning Microscopic 3D images and orthogonal views of *S.oneidensis* MR‐1 WT (A and C) and high c‐di‐GMP strain MR‐1/pYedQ_2_ (B and D) respectively, showing biofilm formation in flow cells after 24 h of growth under hydrodynamic conditions. (White scale bar = 100 μm.).

**Fig. 2:**
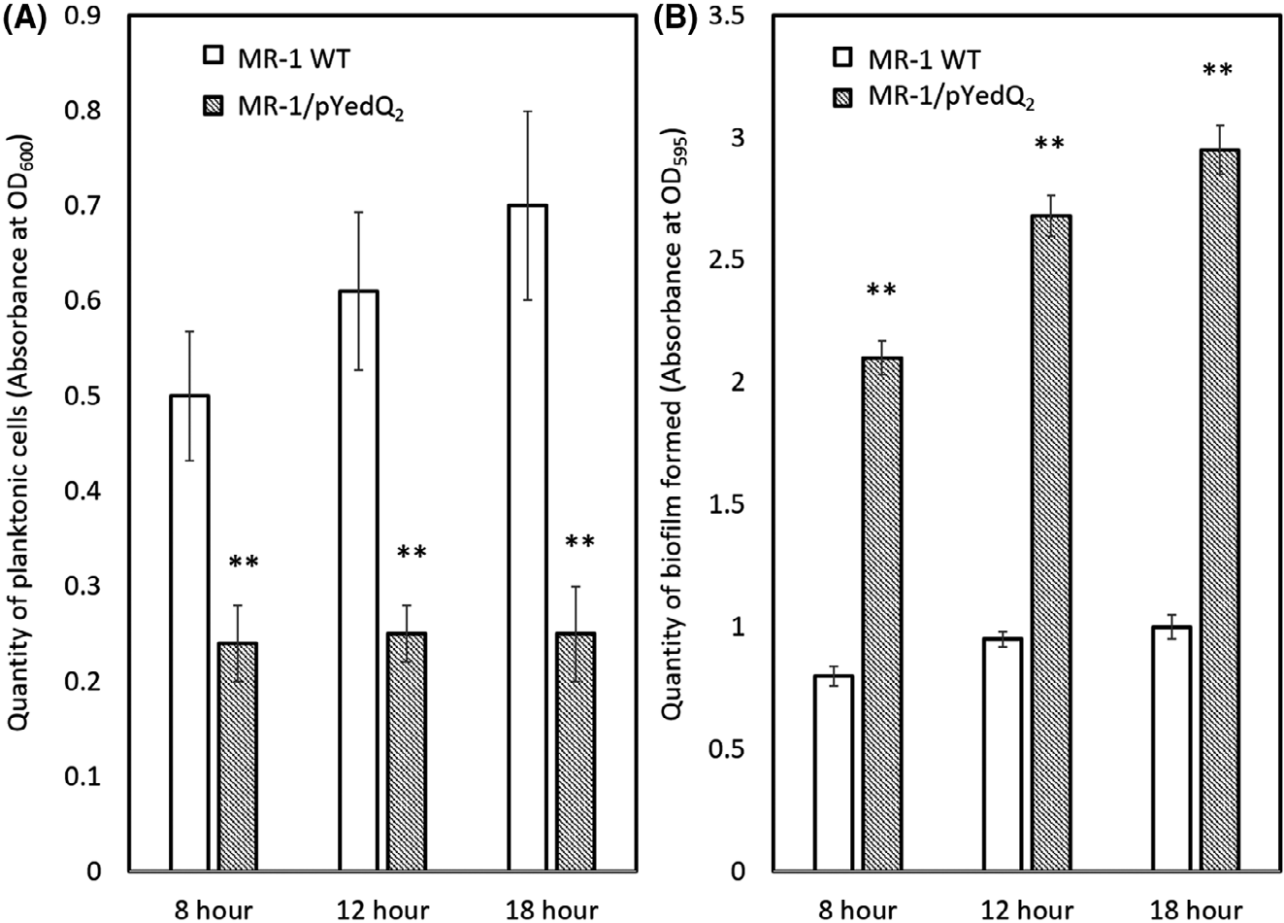
Bar chart of (A) planktonic cells quantity and (B) biofilm formation assay at 8, 12 and 18 h of growth of *S. oneidensis* MR‐1 WT and MR‐1/pYedQ_2_ in 96‐well plate measured using absorbance at OD600 and OD595 respectively. Data are presented as means with standard deviation, where ** indicates statistical significance (*t*‐test, *P*‐value < 0.005, *n* = 40).

MR‐1/pYedQ_2_ strain exhibited a 30% reduction (*t*‐test *P*‐value < 0.05, *n* = 5) in swimming motility (Fig. S1a) and formed thicker biofilm in a flow cell under hydrodynamic condition (Fig. 1) as compared to the WT. This is in good agreement with previous studies where the increase in c‐di‐GMP impeded cell motility in bacteria including *E. coli, Pseudomonas aeruginosa* and *Vibrio cholerae* (Wolfe and Visick, 2008). Under static conditions, MR‐1/pYedQ_2_ had a comparable growth rate to the WT, but the bulk of MR‐1/pYedQ_2_ cells tended to form biofilms while the WT cells preferred to remain in planktonic state of growth (Fig. S2b and Fig. 2). Such preference of MR‐1/pYedQ_2_ towards biofilm state of growth may be attributed to the increase in production of c‐di‐GMP which suppressed flagellar activity and possibly induced surface adhesive proteins such as BpfA and favours biofilm formation (Thormann *et al*., 2005; Cao *et al*., 2011; Zhou *et al*., 2015). Moreover, we measured the intracellular c‐di‐GMP concentration and found that MR‐1/pYedQ_2_ (159.43 pmol c‐di‐GMP/mg cell biomass) has approximately ninefold more c‐di‐GMP than the MR‐1 WT (17.33 pmol c‐di‐GMP/mg cell), verifying that MR‐1/pYedQ_2_ indeed had a higher level of c‐di‐GMP as compared to the WT. These results indicate that we have successfully constructed a high c‐di‐GMP *S. oneidensis* MR‐1 strain, containing the pYedQ_2_ plasmid, which exhibits key characteristics such as lowered swimming motility, greater biofilm growth and increased intracellular c‐di‐GMP concentration. Although previous studies have demonstrated that the *yedQ* gene (from *E. coli*) is active in other types of bacteria such as *Burkholderia cenocepacia* and *Pseudomonas aeruginosa* (Chen *et al*., 2015; Fazli *et al*., 2015), our work is the first study to provide strong evidence that *yedQ* is active in MR‐1, which enabled us to investigate how increased intracellular c‐di‐GMP can influence other cellular functions in MR‐1.

### Transcriptomic analysis of *S. oneidensis* MR‐1 WT vs high c‐di‐GMP strain MR‐1/pYedQ_2_

To elucidate the physiological differences between *S. oneidensis* MR‐1 WT and the MR‐1/pYedQ_2_ strain with high c‐di‐GMP, total RNA from the cells harvested at the mid‐log phase of respective cultures was extracted and sequenced. A total of 4588 genes were transcribed and among them, 446 were significantly different in respect to expression (i.e. Log2 ratio > 1 and < −1) at the transcript level in the MR‐1/pYedQ_2_ strain as compared to the WT (*P*‐value < 0.00001; Fig. S1). Among the up‐regulated genes in the high c‐di‐GMP strain MR‐1/pYedQ_2_, the genes encoding the five primary protein components of the Mtr pathway responsible for EET had significantly greater expression at the transcript level as compared with the WT (Table 1).

**Table 1:** Transcriptomic data of RNA expression of EET protein components in *S. oneidensis* high c‐di‐GMP strain MR‐1/pYedQ_2_ as compared to WT.

Gene	Locus tag	Gene description	Normalized *P*‐value	Log2 ratio
*omcA*	SO1779	Extracelllular iron oxide respiratory system surface decaheme c‐type cytochrome component OmcA	2.244 × 10^−12^	3.728
*mtrC*	SO1778	Extracellular iron oxide respiratory system surface decaheme c‐type cytochrome component MtrC	6.693 × 10^−10^	2.796
*mtrA*	SO1777	Extracelllular iron oxide respiratory system periplasmic decaheme c‐type cytochrome component MtrA	3.203 × 10^−10^	2.241
*cymA*	SO4591	Membrane anchored tetraheme c‐type cytochrome CymA	1.165 × 10^−08^	2.202
*mtrB*	SO1776	Extracellular iron oxide respiratory system outer membrane component MtrB	1.489 × 10^−09^	1.684

The Mtr pathway consists of 5 primary components, namely the porin protein MtrB, and the *c*‐type cytochromes CymA, MtrA, MtrC and OmcA (Shi *et al*., 2007). It is one of the most well‐studied EET chain modules and is essential in cellular respiration using extracellular insoluble minerals, for example hematite, as terminal electron acceptors (Ng et al., 2013a, 2013b,2013a, 2013b). The Mtr pathway plays a significant role in bioremediation, converting environmental contaminants to their more insoluble and less harmful forms. Examples include the reduction of Cr(VI) _(aq)_ to Cr(III) _(s)_, U(VI) _(aq)_ to U(IV) _(s)_, Tc(VII)O_4_^‐^_(aq)_ to Tc(IV)O_2(s)_ and Ag^+^_(aq)_ to Ag(0) _(s)_ (Lovley *et al*., 1991; Marshall *et al*., 2008; Belchik *et al*., 2011; Ng et al., 2013a, 2013b,2013a, 2013b). The Mtr pathway, which confers *S. oneidensis* the ability to perform EET, also plays an important role in bioelectricity generation in microbial fuel cells (Harris *et al*., 2012; Okamoto *et al*., 2012).

As post‐transcriptional regulatory processes in cells are highly prevalent, steady‐state transcript abundances cannot be used to accurately predict protein abundances (Vogel and Marcotte, 2012). With the significant increase in RNA expression related to Mtr‐pathway, we are interested to know whether there are actual phenotypic difference between cells of MR‐1 WT and MR‐1/pYedQ_2_, for example in the amount of functional Mtrpathway proteins and their effectiveness in EET. Fe(III) reduction was undertaken using resting cells assay, which revealed that cells of high c‐di‐GMP strain MR‐1/pYedQ_2_ were able to reduce Fe(III) close to two times faster (21.58 vs 11.88 pM of Fe(III)/h cell) than the WT (Fig. S3). The increase in the Fe reduction rate was most likely caused by the increase in the amount of proteins involved in the EET pathways, but the possibility of an increased in EET efficiency of the proteins can not be ruled out.

**Fig. 3:**
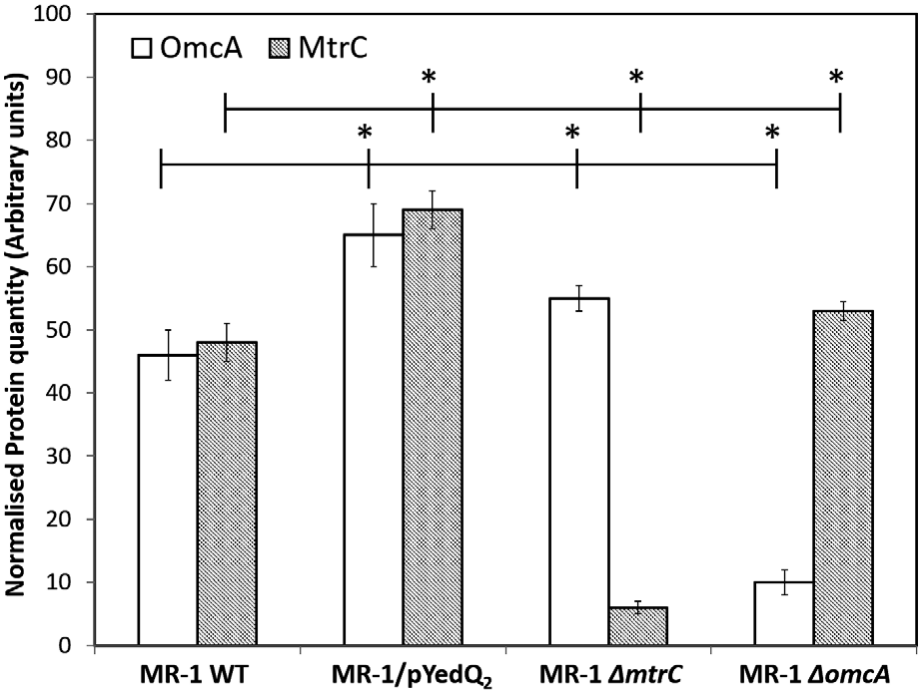
Western blot quantification of outer‐membrane c‐type cytochrome MtrC and OmcA in *S. oneidensis* MR‐1 WT and high c‐di‐GMP strain MR‐1/pYedQ_2_, with mutant lacking MtrC (e.g. Δ*mtrC*) and OmcA (e.g. Δ*omcA*) as negative control. Numerical quantification is derived from proportion of dark pixels to light pixels calculated using ImageJ. Data is presented as means with standard deviation, where * indicates statistical significance (*t*‐test, *P*‐value < 0.05, *n* = 3).

To further verify this observation, we performed western blot analysis to determine the relative amount of MtrC and OmcA proteins in WT and high c‐di‐GMP strain, using MR‐1 mutant strains lacking gene encoding the protein MtrC or OmcA, that is *∆mtrC* and *∆omcA*, as negative controls. A higher amount of outer membrane *c*‐type cytochrome MtrC and OmcA in the high c‐di‐GMP strain MR‐1/pYedQ_2_ was detected compared with WT (Fig. 3). Interestingly, there seemed to be a homeostatic compensation effect where there was an increased expression of OmcA in the Δ*mtrC* mutant and MtrC in the Δ*omcA* mutant as compared to the WT.

Our results suggest that there was an increase in MtrC and OmcA in MR‐1/pYedQ_2_ as compared to WT cells. These results are in agreement with a previous study where it was found that elevated intracellular c‐di‐GMP promotes bioelectricity generation in microbial fuel cells (Liu *et al*., 2015). While the previous study attributed the increase in bioelectricity generation to thicker biofilm formation (i.e. more cells) on the electrode contributing to higher electron transfer, it is apparent from our study that cells with elevated c‐di‐GMP have, on average, a higher amount of *c*‐type cytochromes per cells, making the cells ‘more conductive’ as compared to cells with lower c‐di‐GMP (i.e. MR‐1 WT).

While we have shown that an elevated intracellular concentration of c‐di‐GMP can influence the production of cytochrome MtrC and OmcA in MR‐1, little is known about the expression of cytochromes at the single‐cell level and its nature of distribution across the cell population.

### Semi‐quantitative analysis of c‐type cytochromes using SCRM

Single‐cell Raman microspectroscopy (SCRM) is a vibrational microspectroscopy which can be used to study single bacterial cells by measuring molecular vibrational modes of all biomolecules in a cell. Single‐cell Raman spectra (SCRS) obtained from SCRM can be regarded as label‐free biochemical fingerprints of single cells which reveal phenotypic and intrinsic information of individual cells (Huang *et al*., 2004). To further understand the influence of c‐di‐GMP regulation on cytochrome expression in *S. oneidensis*, we obtained SCRS of MR‐1 WT, high c‐di‐GMP strain MR‐1/pYedQ_2_ and double cytochrome deletion mutant Δ*mtrC*Δ*omcA,* and semi‐quantitatively compare the level of cytochromes. In each case, 100 SCRS were used for the analysis.

**Fig. 4:**
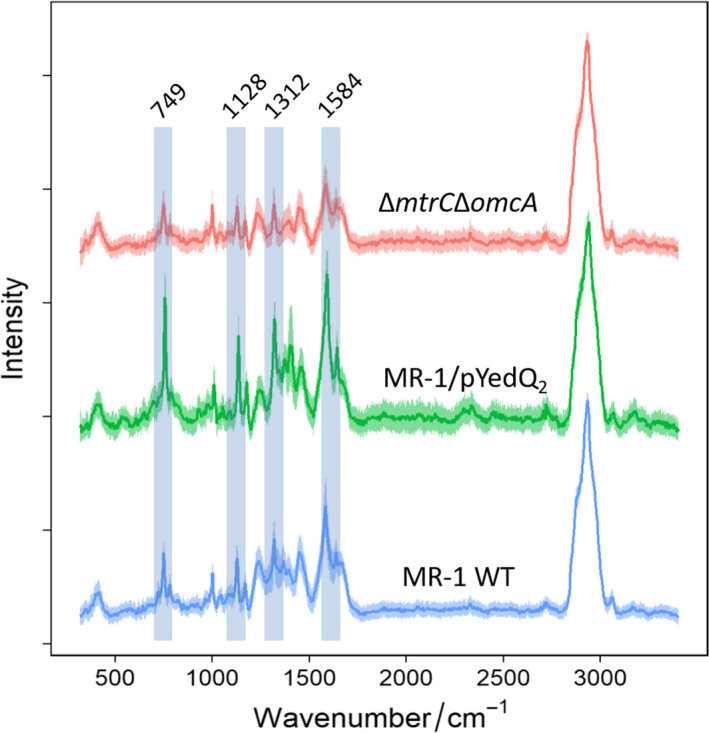
SCRS of *S. oneidensis* MR‐1 WT, high c‐di‐GMP strain MR‐1/pYedQ_2_ and double cytochrome deletion mutant Δ*mtrC*Δ*omcA*, containing Raman signature bands of c‐type cytochrome at 749, 1128, 1312 and 1584 cm^−1^.

**Fig. 5:**
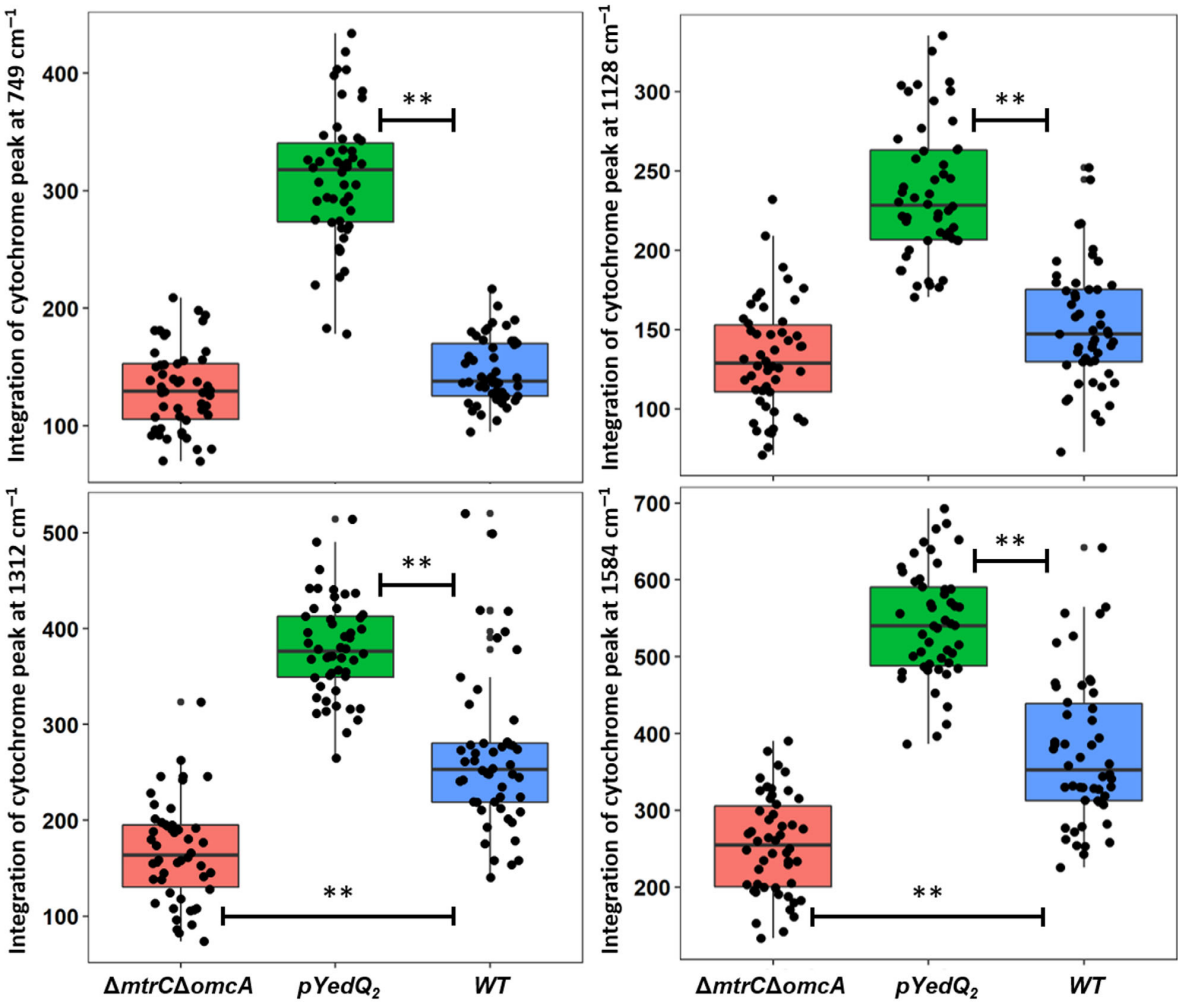
Box plots of single‐cell band integration show the statistical distributions of concentrations of intracellular *c*‐type cytochrome in MR‐1 WT, high c‐di‐GMP strain MR‐1/pYedQ_2_ and MR‐1 Δ*mtrC*Δ*omcA*, a mutant lacking both outer membrane MtrC and OmcA. The rectangle in the box plots represents the second and third quartiles with a line inside representing the median. The lower and upper quartiles are drawn as lines outside the box. ** indicates statistical significance (one‐way ANOVA, *P*‐value < 0.005, *n* = 100).

The *c*‐type cytochrome signature bands at 749 (pyrrole breathing mode), 1128 (ν(CN) stretching vibrations), 1312 (δ(CH) deformations) and 1584 cm^−1^ (ν(CC) skeletal stretches) were observed in cells of MR‐1 WT, MR‐1/pYedQ_2_ and MR‐1 Δ*mtrC*Δ*omcA* (Fig. 4). These four Raman bands at 749, 1128, 1312, and 1584 cm^−1^ are typical *c*‐type cytochrome spectra according to previous reports (Xu *et al*., 2017). By integrating the individual Raman bands of *c*‐type cytochrome, relative concentrations of intracellular *c*‐type cytochrome within the different cells were calculated. All four *c*‐type cytochrome bands were significantly greater in cells of MR‐1/pYedQ_2_ than those of WT (*P* < 0.005; Fig. 5). This is in good agreement with our previous results from the western blot, transcriptomic analysis and iron reduction experiment, showing that MR‐1/pYedQ_2_ cells contain higher amount of *c*‐type cytochromes.

Interestingly, the cytochrome bands at 1312 cm^−1^ and 1584 cm^−1^ were lower in cells of Δ*mtrC*Δ*omcA* as compared to those of WT, while such differences were insignificant at 749 and 1128 cm^−1^ (Fig. 5). Although the cells of Δ*mtrC*Δ*omcA* lacked the outer membrane *c*‐type cytochrome MtrC and OmcA, it is not entirely obvious that the overall amount of cytochrome in Δ*mtrC*Δ*omcA* will be much lower than WT. There are many different *c*‐type cytochromes that have been identified in MR‐1, with previous report of 42 possible *c*‐type cytochrome genes in its genome (Meyer *et al*., 2004), suggesting that the removal of MtrC and OmcA may not significantly decrease the amount of total *c*‐type cytochromes in the cell. Moreover, homeostatic compensation mechanisms in cells of Δ*mtrC*Δ*omcA* may result in possible increase in expression of other *c*‐type cytochromes with overlapping functions, such as MtrF (Golitsch *et al*., 2013). Such homeostatic compensation was also observed in the western blot where cells of Δ*mtrC* had a slightly higher expression of OmcA and cells of Δ*omcA* had a slightly higher expression of MtrC, as compared to cells of WT. These might explain why only some of the signature *c*‐type cytochrome Raman bands were significantly lower in cell of Δ*mtrC*Δ*omcA* as compared to MR‐1 WT, which reflects the possible scenario where Δ*mtrC*Δ*omcA* had slightly lower total amount of *c*‐type cytochrome as compared to WT.

In this study, SCRM was used to discriminate the phenotypic changes of *S. oneidensis*, specifically by the amount of cytochromes in cells of MR‐1 WT, high c‐di‐GMP strain MR‐1/pYedQ_2_ and double cytochrome deletion strain Δ*mtrC*Δ*omcA*. As its name suggests, SCRM provides accurate chemical information at single‐cell level, allowing variation and dynamics of a specific phenotype in a population to be investigated. This is in contrast to bulk analysis which provides a mean or average measurement value with no information on the variation and deviation of individuals within the sampled population. The detection range of SCRS was from 400 to 3600 cm^−1^, covering most biomolecular vibrations of single cells. *c*‐type cytochromes will generate a resonance Raman effect as the heme group absorbs visible light around 530 nm when using an incident Raman laser at 532 nm. Thus, the resonance effect selectively enhances the signals of *c*‐type cytochromes as compared to other peaks in the spectrum (Pätzold et al., 2008). SCRM can be used to perform non‐destructive and semi‐quantitative analysis of biomolecules such as c‐type cytochrome on live cells at single‐cell spatial resolution. However, for accurate quantification, other chemical analysis tools, such as proteomics are needed, although it is difficult to measure at the single‐cell level.

**Fig. 6:**
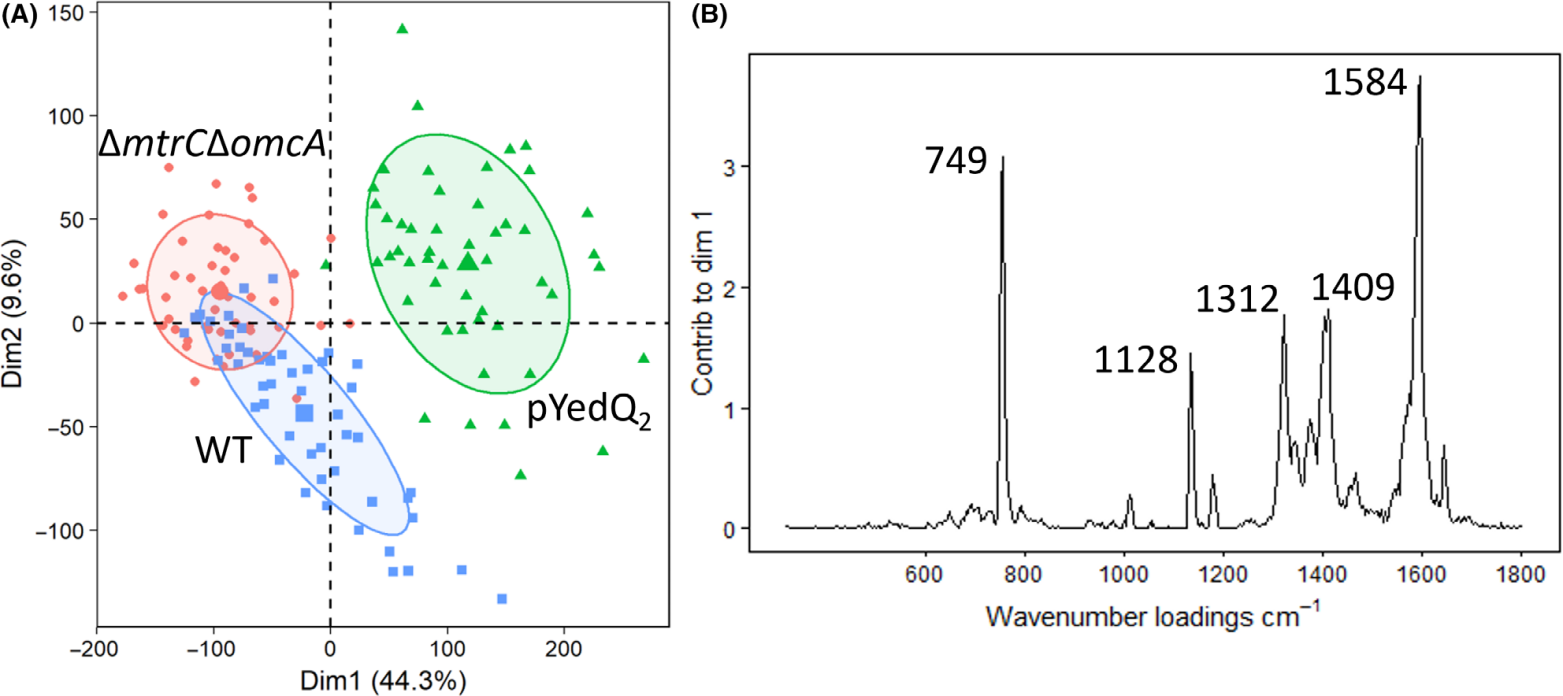
PCA analysis of SCRS of MR‐1 WT, high c‐di‐GMP strain MR‐1/pYedQ_2_ and Δ*mtrC*Δ*omcA*, a mutant lacking both outer membrane MtrC and OmcA, showing (A) a PCA plot along the first two dimensions of the principal components and (B) a loading plot of Raman wavenumbers along dimension 1 with the highest contribution by *c*‐type cytochrome signature bands.

Besides quantification of some known features in the spectrum (the cytochromes in this case), dimension‐reducing techniques are often desired to resolve the complex Raman dataset as a result of more than 1,500 Raman bands present in one spectrum. Here, unsupervised principal component analysis (PCA) was applied to reduce the high‐dimensional Raman dataset and reveal key information responsible for single‐cell variances. PCA plot along the first two dimensions of the principal components clearly revealed three clusters of single cells of MR‐1 WT, high c‐di‐GMP strain MR‐1/pYedQ_2_ and MR‐1 Δ*mtrC*Δ*omcA*, respectively (Fig. 6A). It suggests that the three strains have distinctive phenotypic profiles. As the separation was most evident along dimension 1, the contributions of Raman wavenumbers along dimension 1 (Fig. 6B) were plotted. The highest contributions were attributed to 749, 1128, 1312 and 1584 cm^−1^, which are consistent with the peak positions of *c*‐type cytochrome (Xu *et al*., 2017), suggesting the level of c‐type cytochrome in single cells are the major difference in these cell types. Interestingly, a peak (of unknown identity) at 1409 cm^−1^ also contributes significantly to the classification of the strains warranting further investigations. As the dimension 1 of the PCA explains 44.3% of the single‐cell variances (Fig. 6B), it suggests that the cytochromes accounted for the most significant differences within single cells, as well as among three strains.

## Conclusion

Employing metal reduction assay, transcriptomic analysis, western blotting and single‐cell Raman spectroscopy, we showed that an elevated intracellular c‐di‐GMP concentration increased the expression of c‐type cytochromes in *S. oneidensis* MR‐1. The results demonstrated for the first time that c‐di‐GMP regulation system indirectly or directly positively regulates the expression of cytochromes including those involved in the extracellular electron transport such as CymA, MtrA, MtrC and OmcA of the Mtr pathway. This result provides molecular insight into a new mechanism underpinning the extracellular electron transfer in bulk biofilms and opens new opportunities in exploiting the functionality of biofilms critical in industrial processes involving bioelectricity generation and other biological redox reactions.

## Experimental procedures

### Chemicals, bacterial strains and growth conditions

Chemicals used in this study are sourced from Sigma Aldrich (United Kingdom) without further modification unless otherwise stated. Bacterial strains and plasmids used in this study are described in Table 2. Stock cultures were maintained in Lysogeny Broth (LB) medium with 20% glycerol at −80°C. The bacterial cells were grown in LB medium or modified M1 medium under aerobic or anaerobic conditions. The modified M1 medium (Myers and Nealson, 1988) was used for all experiments and contained: 28 mM NH_4_Cl, 1.34 mM KCl, 4.4 mM Na_2_HPO_4_, 1.5 mM Na_2_SO_4_, 0.7 mM CaCl_2_, 1 mM MgCl_2_, 5 mM PIPES, a vitamin mixture (1 l of medium contains 0.02 mg Biotin, 0.02 mg folic acid, 0.1 mg pyridoxine HCl, 0.05 mg thiamine HCl, 0.05 mg riboflavin, 0.05 mg nicotinic acid, 0.05 mg dl‐pantothenic acid, 0.05 mg p‐aminobenzoic acid, 0.05 mg lipoic acid, 2 mg choline chloride, 0.01 mg vitamin B12) and trace elements (1 l of medium contains 10 mg FeCl_2_ 4H_2_O, 5 mg MnCl_2_ 4H_2_O, 3 mg CoCl_2_ 4H_2_O, 2 mg ZnCl_2_, 0.5 mg Na_2_MoO_4_ 4H_2_O, 0.2 mg H_3_BO_3_, 1 mg NiSO_4_ 6H_2_O, 0.02 mg CuCl_2_ 2H_2_O, 0.06 mg Na_2_SeO_3_ 5H_2_O, 0.08 mg Na_2_WO_4_ 2H_2_O). The medium was adjusted to a pH of 7.0 by addition of 1 M NaOH and 20 mM sodium lactate was used as an electron donor and the sole carbon source. For growth under anaerobic conditions, the medium containing 10 mM ferric nitrilotriacetate (Fe(III)‐NTA) as an electron acceptor was bubbled with nitrogen gas for 5 min to remove O_2_ from the headspace and the headspace in the culture was less than 3% of the total volume.

**Table 2:** Bacterial strains and plasmids used in this study.

Strains/Plasmid	Description	References
Strains		
*S. oneidensis* MR‐1		
Wild‐type (WT)	Manganese‐reducing strain; wild‐type	Myers and Nealson (1988)
MR‐1 *ΔmtrC*	Deletion derivative of SO1778 (*mtrC*) gene within MR‐1 WT	Marshall *et al*. (2006)
MR‐1 *ΔomcA*	Deletion derivative of SO1779 (*omcA*) gene within MR‐1 WT	Marshall *et al*. (2006)
MR‐1 *ΔmtrCΔomcA*	Deletion derivation of SO1779 (*omcA*) gene within MR‐1 Δ*mtrC*	Marshall *et al*. (2006)
MR‐1/pYedQ_2_	MR‐1 strain with elevated intracellular c‐di‐GMP level	This study
*E. coli*		
DH5α	Strain used for standard DNA manipulations	Zhang *et al*. (2014)
HB101/pRK600	Strain habouring pRK600 and used as helper cell for conjugation	Chen *et al*. (2015)
DH5α/pYedQ_2_	Strain habouring pYedQ_2_ and used as donor cell for conjugation	Chen *et al*. (2015)
Plasmid		
pYedQ_2_	Gm^r^; pBBR1MCS‐5 carrying the YedQ gene	Fazli *et al*. (2015)
pRK600	Cm^r^; oriColE1 RK2‐Mob^+^ RK2‐Tra^+^; helper vector for conjugation	Kessler *et al*. (1992)

### Construction of high c‐di‐GMP strain of *S. oneidensis*

The *yedQ* (NCBI Gene ID: ID: 7149712, previously known as *yhcK*) gene from *Escherichia coli* IAI39 was cloned from the pYedQ plasmid (Ausmees *et al*., 2001; Chen *et al*., 2015) into the *HindIII/BamHI* side of pBBR1MCS‐5 plasmid vector to make the pYedQ_2_ plasmid (Fazli *et al*., 2015). *E. coli* DH5α was transformed with the pYedQ_2_ plasmid via heat shock (Hanahan *et al*., 1991). The *S. oneidensis* strain with an elevated c‐di‐GMP level (i.e. MR‐1/pYedQ_2_) was constructed via plate mating (i.e. tri‐parental conjugation) using the following strains at mid‐logarithmic growth stage: *S. oneidensis* MR‐1 (recipient), *E. coli* HB101/pRK600 (helper) and *E. coli* DH5α/pYedQ_2_ (donor). Further experimental details on plate‐mating can be found in Supplementary Information (Text S1). Agarose gel electrophoresis using Mini‐Sub^®^ Cell GT Cell (Bio‐Rad, Hercules, CA, USA) was run with the plasmid extracted from the selected mutant as a confirmation that the selected mutant indeed contains the pYedQ_2_ plasmid. All swim plate experiments were done in 0.3% LB agar plates at 30°C for 24 h with five replicates for each sample (Fig. S1). CFU counts using LB agar plates with and without 50 µg ml^−1^ gentamicin of MR‐1/pYedQ_2_ culture grown for 12, 24 and 48 h showed insignificant differences (*t*‐test *P*‐value < 0.05, *n* = 3). No growth was observed for MR‐1 WT on LB agar plates with 50 µg ml^−1^ gentamicin at 12, 24 and 48 h.

### Cell growth and static biofilm assay

Cultures of *S. oneidensis* MR‐1 WT and MR‐1/pYedQ_2_ at mid‐logarithmic growth stage were inoculated into 96‐well plates containing 200 μl of LB medium in each well. Forty replicates of MR‐1 WT, MR‐1/pYedQ_2_ and 16 replicates of abiotic control were setup in each of the 96‐well plates. To measure planktonic cell growth, the plates were left to incubate in Infinite^®^ 200 PRO plate reader (Tecan, Switzerland) at 30°C with periodic shaking, and OD_600_ readings were taken every 10 min over 20 h. To measure the biofilm growth, the cultures were allowed to grow for 8, 12 and 18 h in 96 well‐plates, respectively, before the static biofilm assay was done in accordance to the microtiter plate biofilm assay protocol (Merritt *et al*., 2005) using optical density measurements at wavelength of 595 nm (OD_595_). Briefly, each well was drained to remove planktonic cells and the remaining adherent biomass, i.e., biofilms, were washed with 0.9% NaCl solution and stained with 1% Crystal Violet solution. The stained biofilm samples were washed with 0.9% NaCl to remove excess crystal violet and then dissolved in ethanol, where samples with thicker biofilm results in higher OD_595_ measurements. Colony‐forming unit (CFU) for both planktonic and biofilm cells of MR‐1 WT and MR‐1/pYedQ_2_ was counted to verify the OD results. Differences between mean CFU were determined by *t*‐test (*n* = 40, *P*‐value < 0.005).

### C‐di‐GMP extraction and quantification

C‐di‐GMP was extracted and quantified as previously described with modifications (Wu *et al*., 2015a, 2015b,2015a, 2015b; Hu *et al*., 2017). Cultures of *S. oneidensis* MR‐1 WT and MR‐1/pYedQ_2_ were grown to mid‐logarithmic stage, when an aliquot of 2 ml was taken from each culture and centrifuged at 10 000 *g* for 3 min. The supernatant was discarded, and the cell pellets were washed with and resuspended in 2 ml of 1 mM ice‐cold ammonium acetate. The respective cell suspensions were passed through a 20‐gauge needle five times to increase cell separation and improve effectiveness of washing. After centrifugation, the cell pellets were suspended in 2 pellet volume of ice‐cold solution comprising of acetonitrile, methanol and water at 2:2:1 ratio. The resultant cell suspensions were lysed using Q125‐220 125 watt Sonicator (QSonica, Connecticut, USA) with a 3.175 mm diameter probe at 500 J with 10s–10s sonication‐cooling time ultrasound regime for 5 min in an ice bath. The resultant lysates were centrifuged, and the supernatants were retained. The volume of the supernatants was reduced initially at 4°C and 5000 Pa by temperature‐controlled speedvac (Thermo Scientific, Singapore) to around 1 ml, while the remaining solutions were subjected to lyophilization (Labconco, Kansas City, MO, USA) at 500 Pa and −80°C. The lyophilized samples were then suspended in 100–200 μl of 1 mM ammonium acetate, sonicated for 10 min in ice bath and centrifuged for 5 min at 10 000 rpm. The supernatants containing c‐di‐GMP were transferred to glass inserts for analysis using liquid chromatography as previously described (Kuchma *et al*., 2010). Experiments were conducted in triplicates and *t*‐test used to determine differences between means (*P*‐value < 0.05).

### Iron reduction and quantification

Cultures of *S. oneidensis* MR‐1 WT and MR‐1/pYedQ_2_ were grown to mid‐logarthimic stage, when an aliquot of 10 ml was taken from each culture 10 000 *g* for 10 min, and the cell pellets were washed three times using 30 mM HEPES buffer. The cells were suspended in the HEPES buffer, and the final OD_600_ of 0.1 were achieved for all suspensions. The cell suspensions were placed in anaerobic glass tubes capped with butyl rubber, with three replicates of each for the WT and high c‐di‐GMP strain, and each setup was bubbled with N_2_ gas for 10 min. Sodium lactate and Fe‐NTA were subsequently added into the set‐up in an anaerobic chamber (Shel Lab, Cornelius, USA) to a final concentration of 20 mM and 10 mM, respectively. Iron was quantified using Ferrozine Assay as previously described (Riemer *et al*., 2004). Experiments were conducted in triplicates and t‐test used to determine differences between means (*P*‐value < 0.05).

### Western blotting

Western blotting was done as previously described (Shi et al., 2006, 2008). Cultures of *S. oneidensis* MR‐1 WT and high c‐di‐GMP strain MR‐1/pYedQ_2_ were grown in LB media (10 ml) till mid‐logarithmic growth stage in a temperature‐controlled shaking incubator (30°C, 200 r.p.m.). The cells were harvested and washed three‐times using 1 × PBS buffer. The cell pellet was suspended in 200 μl of SDS‐PAGE loading buffer, heated for 10 min at 70°C, cooled to room temperature, and incubated with 2 μl of DNase1 for 10–15 min at 37°C. Total protein concentration was tested and samples normalized to 1 μg μl^−1^. Samples containing 10 μg of protein were then directly subjected to NuPAGE (8% Tris‐Acetate gels) followed by dry protein transfer onto a nitrocellulose membrane using the iBlot system (InVitrogen, Carlsbad, CA, USA). The membranes were developed using Western blot protocol and antibodies previously described (Shi *et al*., 2008) [primary antibody: Rabbit *IgG* (H + L), dilution 1:3000; secondary antibody: anti‐rabbit horseradish peroxidase, dilution 1:16 000] targeting the protein MtrC and OmcA, and imaged using a Fuji LAS‐1000 system and Image reader for LAS‐1000 Pro (Fujifilm, Tokyo, Japan). Data analysis of the western blot gel image was done using ImageJ (Schneider *et al*., 2012). Experiments were conducted in triplicates and t‐test used to determine differences between means (*P*‐value < 0.05).

### RNA extraction and sequencing analysis

RNA extraction and sequencing analysis were conducted as previously described (Wu *et al*., 2015a, 2015b, 2016,2015a, 2015b, 2016) on mid‐logarithmic stage culture of MR‐1 WT and MR‐1/pYedQ_2_ using the RNeasy mini Kit (Qiagen, Hilden, Germany), RNase‐free DNase set (Qiagen) and Illumina HiSeq2500 (Illumina, San Diego, CA, USA) according to the manufacturer’s instructions. Experimental details can be found in Supplementary Information (Text S2). A volcano plot (Fig. S1) was used to visualize the distribution and identify around ~ 10% of the total genes with large magnitude change that are also statistically significant, which resulted in the selection criteria of genes (446 out of 4588) with twofold (i.e. 1‐fold Log_2_‐scale) changes in expression and a *t*‐test *P*‐value ≤ 0.00001 to be considered significant in this study.

### Growth of biofilms

Biofilms of *S. oneidensis* MR‐1 WT and the high c‐di‐GMP strain MR‐1/pYedQ_2_ were grown in three‐channel flow cells (channel dimensions, 1 × 4 × 40 mm^3^) using 1/10th‐strength LB medium containing 10 mM lactate continuously supplied through a peristaltic pump at a flow rate of 10 ml h^−1^. The flow system was assembled and sterilized as described previously (Sternberg and Tolker‐Nielsen, 2006). Each flow cell channel was inoculated with 0.3 ml overnight culture (diluted to an OD_600_ of 0.1) using a syringe. After inoculation, the medium flow was stopped for 1 h to allow initial attachment followed by continuous media flow with a flow rate of 10 ml h^−1^. Biofilms samples were stained with LIVE/DEAD BacLight Bacterial Viability Kit (Thermo Fisher Scientific, Waltham, MA, USA) according to the manufacturer’s instructions and viewed using ZEISS LSM 900 with Airyscan 2 confocal laser‐scanning microscope (Carl Zeiss AG, Oberkochen, Germany).

### Single‐cell Raman Microspectroscopy (SCRM)

Single‐cell Raman spectra (SCRS) were obtained with modification as previously described (Song *et al*., 2017; Xu *et al*., 2017). Briefly, cells were harvested by centrifugation, resuspended in Milli‐Q ultrapure water (Sigma Aldrich, UK) and spread onto an aluminium‐coated slide. SCRS of the cells were acquired using an HR Evolution confocal Raman microscope (Horiba Jobin‐Yvon, UK) equipped with a 532 nm neodymium‐yttrium aluminium garnet laser. An objective with a magnification of 100×/NA0.8 was used to focus on single cells, and Raman scattering was detected by a charge‐coupled device (CCD) cooled at −70°C. The spectra were obtained in the range of 400 to 3600 cm^−1^ with a 300 grooves/mm diffraction grating. Acquisition time was 10 s per spectrum with 4.6 mW laser power and 1 μm^2^ laser spot size. For each sample, 100 single cells were measured and analysed.

### Data processing and multivariate analysis of SCRS

All spectra were pre‐processed by cosmic ray correction and polyline baseline fitting using LabSpec 6 (Horiba, United Kingdom). Spectra were normalized by vector normalization of the entire spectral region. Principal component analysis (PCA) was performed under an R environment. Relative concentrations of biomolecules within single‐cells were estimated by integrating individual Raman bands. Box plots showing the quantification distribution were plotted using R. The rectangle in the box plots represents the second and third quartiles with a line inside representing the median. The lower and upper quartiles are drawn as lines outside the box. One‐way ANOVA was used to determine differences between means (*n* = 100, *P*‐value < 0.005).

## Conflict of Interest

The authors declare that the research was conducted in the absence of any commercial or financial relationships that could be construed as a potential conflict of interest.

## Supporting information


**Text S1.** Construction of high c‐di‐GMP strain of *Shewanella oneidensis* MR‐1.
**Text S2.** RNA extraction and sequencing analysis.
**Fig. S1.** A volcano plot of the fold change in expression of genes (log_2_‐scale) versus their significance (*P*‐value in −log_10_‐scale). ~10% of the total genes (446 out of 4588) with more than 2‐fold change (i.e. 1‐fold log_2_‐scale) and *P*‐value less than 0.00001 is considered significant in this study and are demarcated by the red boxes in the plot.
**Fig. S2.** Representative image showing a) differences in swimming motility between S. oneidensis MR‐1 WT and MR‐1/pYedQ_2_, where a significant reduction in swimming motility (30%) of MR‐1/pYedQ_2_ was observed as compared to WT (*t*‐test *P*‐value < 0.05, *n* = 5, black (30%) of MR‐1/pYedQ_2_ was observed as compared to WT (*t*‐test *P*‐value < 0.05, *n* = 5, black compared to WT (white scale bar = 15mm).
**Fig. S3.** Rate of Fe(III) reduction by *S. oneidensis* MR‐1 WT and 2 high c‐di‐GMP strain MR‐1/pYedQ_2_. * indicates statistical significance (*t*‐test *P*‐value<0.05, *n* = 3).
