# Supplementary material for: Elevated intracellular cyclic‐di‐GMP level in Shewanella oneidensis increases expression of c‐type cytochromes
Source: Microb Biotechnol. 2020 Jul 30;13(6):1904–16. doi: 10.1111/1751-7915.13636 (PMC7533324; doi:10.1111/1751-7915.13636)
Supplement: Supplementary file 1 — Text S1. Construction of high c‐di‐GMP strain of Shewanella oneidensis MR‐1. Text S2. RNA extraction and sequencing analysis. Fig. S1. A volcano plot of the fold change in expression of genes (log2‐scale) versus their significance (P‐value in −log10‐scale). ~10% of the total genes (446 out of 4588) with more than 2‐fold change (i.e. 1‐fold log2‐scale) and P‐value less than 0.00001 is considered significant in this study and are demarcated by the red boxes in the plot. Fig. S2. Representative image showing a) differences in swimming motility between S. oneidensis MR‐1 WT and MR‐1/pYedQ2, where a significant reduction in swimming motility (30%) of MR‐1/pYedQ2 was observed as compared to WT (t‐test P‐value < 0.05, n = 5, black (30%) of MR‐1/pYedQ2 was observed as compared to WT (t‐test P‐value < 0.05, n = 5, black compared to WT (white scale bar = 15mm). Fig. S3. Rate of Fe(III) reduction by S. oneidensis MR‐1 WT and 2 high c‐di‐GMP strain MR‐1/pYedQ2. * indicates statistical significance (t‐test P‐value<0.05, n = 3). [file MBT2-13-1904-s001.pdf]

Supplementary Information

Elevated intracellular cyclic-di-GMP level in *Shewanella oneidensis* increases expression of *c*-type cytochromes

Chun Kiat Ng[1,2], Jiabao Xu[1], Zhao Cai[2], Liang Yang[2,#], Ian Thompson[1], Wei E. Huang[1], Bin Cao[2,3]

[1] Department of Engineering Science, University of Oxford, Parks Road, OX1 3PJ, United Kingdom

[2] Singapore Centre for Environmental Life Sciences Engineering, Nanyang Technological University, Singapore

[3] School of Civil and Environmental Engineering, Nanyang Technological University, Singapore

[#] Current address: School of Medicine, Southern University of Science and Technology, China

Co-corresponding Authors:

Wei E. Huang (Tel.: +44 1865 283786. E-mail: wei.huang@eng.ox.ac.uk)

Bin Cao (Fax: +65 6316 7349. Tel: +65 6592 7895. E-mail: bincao@ntu.edu.sg)

First Author: Chun Kiat Ng

Keywords: Cyclic-di-GMP, *Shewanella oneidensis* MR-1, *c*-type cytochrome, Mtr pathway, extracellular electron transfer, single-cell Raman microspectroscopy

Supplementary Text

Text S1

Construction of high c-di-GMP strain of *Shewanella oneidensis* MR-1

YedQ (previously known as *yhcK*) gene from *E. coli* is cloned from the *pYedQ* plasmid in HindIII/BamHI side of pBBR1MCS-5 plasmid vector to make the pYedQ$_2$ plasmid (Chen, et al., 2015). The *S. oneidensis* strain with an elevated c-di-GMP level (i.e. MR-1/pYedQ$_2$) was constructed via plate mating (i.e. tri-parental conjugation) using the following strains at mid-logarimic growth stage: *S. oneidensis* MR-1 (recipient), *E. coli* HB101/pRK600 (helper) and *E. coli* DH5α/pYedQ$_2$ (donor). Recipient, helper and donor cells were grown using LB media overnight in shaking incubator (200 rpm) at 30 °C, 37 °C and 37 °C respectively. Antibiotics such as chloramphenicol and gentamicin were used in cell culturing when necessary. 1 ml of the culture from each of the three strains was washed two times with fresh LB medium, using centrifugation at 10000 x g for 3 minutes. The cell pellets from each of the three strains was resuspended and mixed at 1:1:1 ratio in 1 ml of LB media. Centrifugation was done to obtain a cell pellet containing cells from all three strains in equal proportions. The cell pellet was transferred to a LB agar plate and incubated in 30 °C for 5 hours. The cells were harvested from the plate via washing with 1 ml of 0.9% sodium chloride solution (saline). 100 ul of cells were spread on a LB agar plate containing 20 ug/ml tellurite (to remove *E. coli*) and 60 ug/ml gentamicin (to remove cells without pBBR1MCS-5 plasmid) and kept in incubator at 30 °C until transposon mutant colonies appeared. Agarose gel electrophoresis using Mini-Sub® Cell GT Cell (Bio-Rad, USA) was run with the plasmid extracted from the selected mutant as a confirmation that the selected mutant indeed contains the *pYedQ$_2$* plasmid. All swim plate experiments were done in 0.3% LB agar plates

at 30 °C for 24 hours with five replicates for each sample and t-test was used to analyse the data, where p-value < 0.05 indicates significance.

Text S2

RNA extraction and sequencing analysis

S. oneidensis MR-1 WT and MR-1/pYedQ$_2$ cultures were grown in LB medium aerobically for 8 h at 30 °C with shaking at 200 rpm. This was followed by RNA isolation using the RNeasy mini Kit (Qiagen, Germany), with the DNA being depleted using RNase-free DNase set (Qiagen, Germany) according to the manufacturer's instructions. The concentration of RNA in each sample was determined using Nanodrop 2000 spectrophotometer (Thermo Scientific, USA) and further confirmed using Qubit 2.0 Fluorometer (Life Technologies, Germany). All RNA samples were promptly stored at -80 °C until further use.

For each strain, three biological replicates and three analytical replicates for each biological replicate were subjected for RNA sequencing in this experiment. A total of 18 RNA samples from MR-1 WT and MR-1/pYedQ$_2$ were sequenced by the sequencing facilities in the Singapore Centre on Environmental Life Sciences Engineering (SCELSE). Quality of the RNA samples was determined using the Quant-iT™ RiboGreen® RNA Assay Kit (Invitrogen) and Quant-iT™ PicoGreen® dsDNA Assay Kit (Invitrogen) on a Bioanalyzer RNA 6000 Nano Chip (Agilent). Next-generation sequencing library was prepared by following the TruSeq RNA Sample Preparation v2 protocol (Illumina) with modifications: The mRNA purification step was omitted and instead, 200 ng of total RNA was directly added to the elute-fragment-prime step. The PCR amplification step, which selectively enriches for library fragments that have adapters ligated on both ends, was performed according to the manufacturer's recommendation but the number of amplification cycles was reduced to 12. Each library was uniquely tagged with one of Illumina's TruSeq LT RNA barcodes to allow library pooling for sequencing. Library quantitation was performed using Invitrogen's Picogreen assay and

the average library size was determined by running the libraries on a Bioanalyzer DNA 1000 chip (Agilent). Library concentration was normalized to 2 nM and the concentration was validated by qPCR on a ViiA-7 real-time thermocycler (Applied Biosystems), using qPCR primers recommended in Illumina's qPCR protocol and Illumina's PhiX control library as a standard. Libraries were then pooled at equal volumes and sequenced in two lanes of an Illumina HiSeq2500 rapid run at a final concentration of 7.2 pM and a read-length of 101 bp paired-end.

The Illumina reads were functionally assigned by mapping against the published genome of *S. oneidensis* MR-1 (Heidelberg, et al., 2002) using the CLC Genomics Workbench (CLC bio, Aarhus, Denmark) (Mortazavi, et al., 2008). The mRNA reads were normalized using the RPKM (reads assigned per kilo-base of target per million mapped read) method. A t-test using RPKM normalized values with FDR (False Discovery Rate) p-value correction were performed.

Supplementary Figure

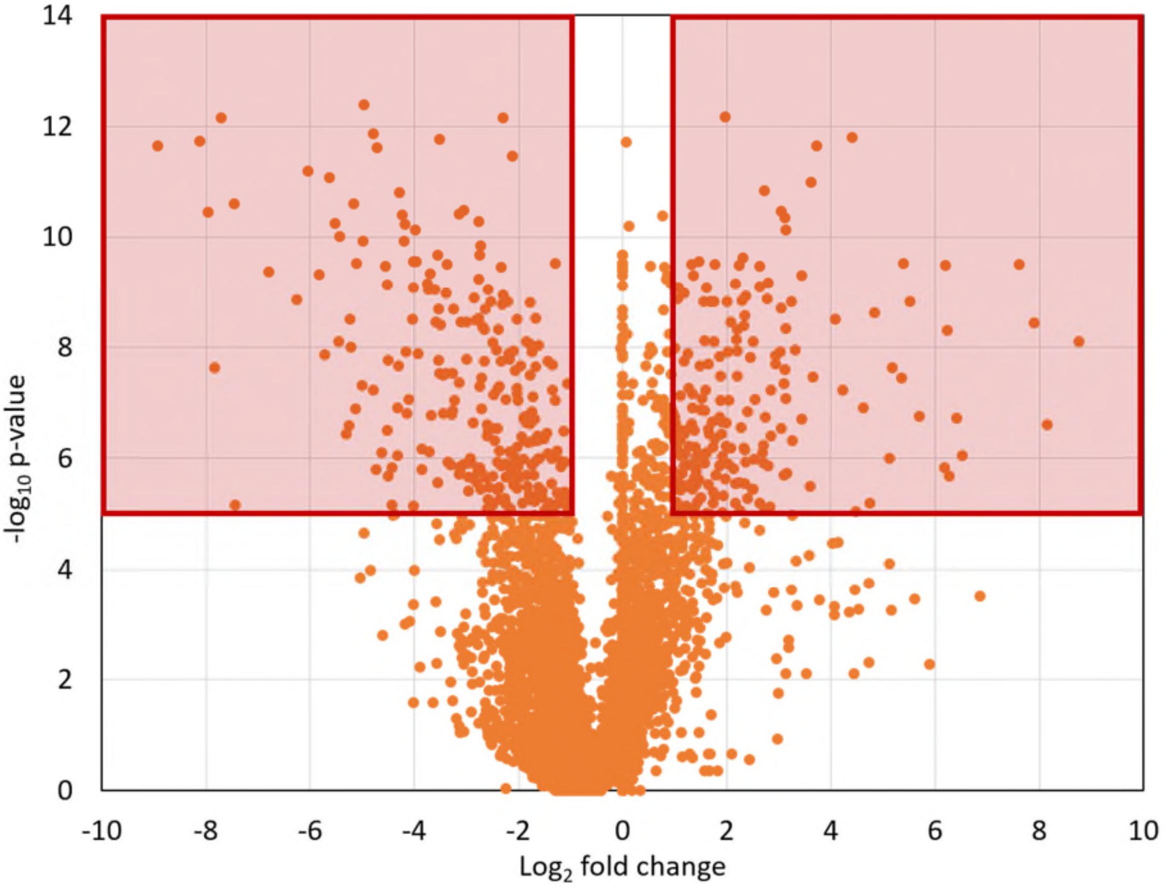

Fig. S1. A volcano plot of the fold change in expression of genes (log_2-scale) versus their significance (p-value in $-log_{10}$-scale). ~10% of the total genes (446 out of 4588) with more than 2-fold change (*i.e.* 1-fold log2-scale) and p-value less than 0.00001 is considered significant in this study and are demarcated by the red boxes in the plot.

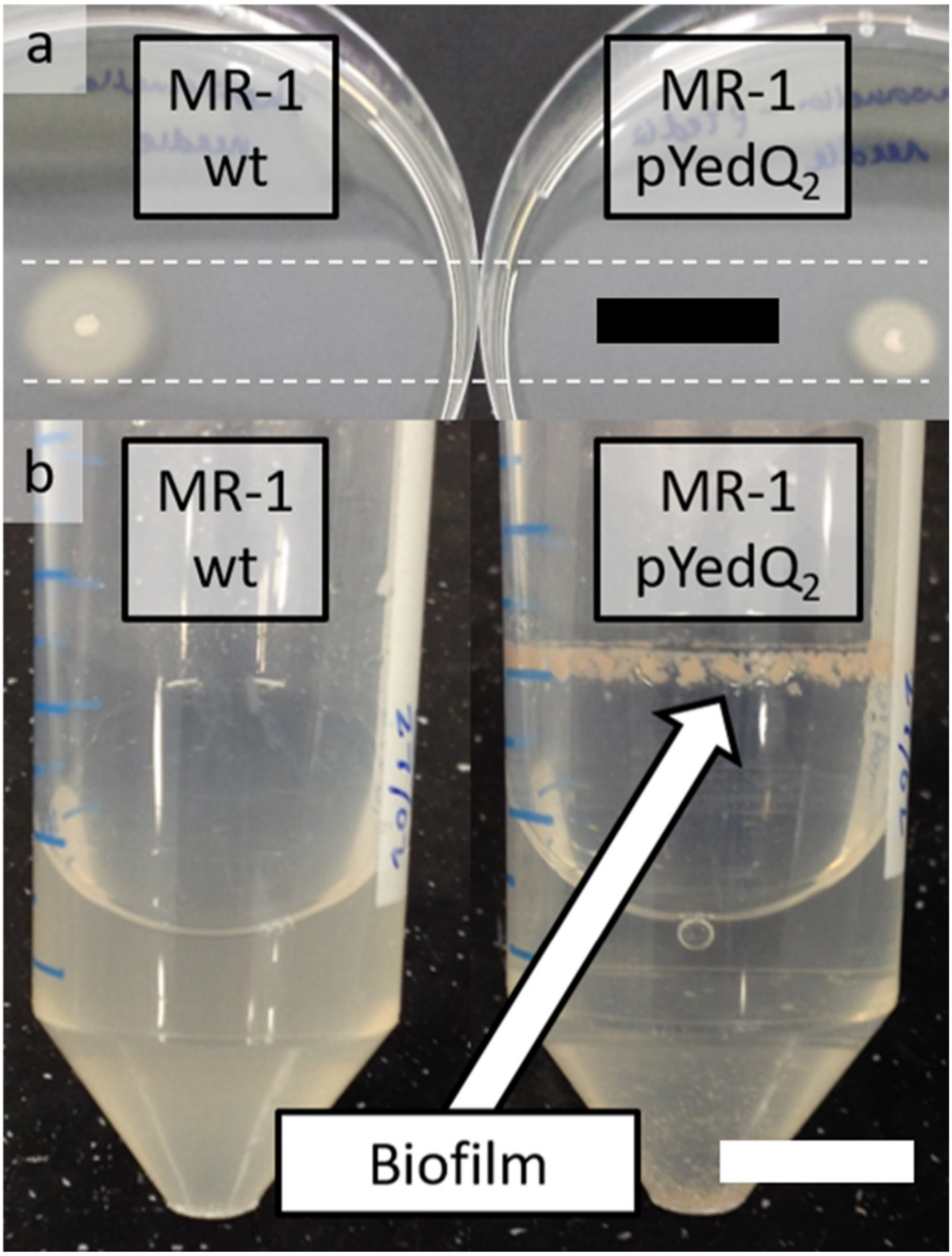

Figure S2. Representative image showing a) differences in swimming motility between S. oneidensis MR-1 WT and MR-1/pYedQ$_2$, where a significant reduction in swimming motility (30%) of MR-1/pYedQ$_2$ was observed as compared to WT (t-test p-value < 0.05, $n = 5$, black scale bar = 50mm), and b) preference for biofilm formation in cells of MR-1/pYedQ$_2$ as compared to WT (white scale bar = 15mm).

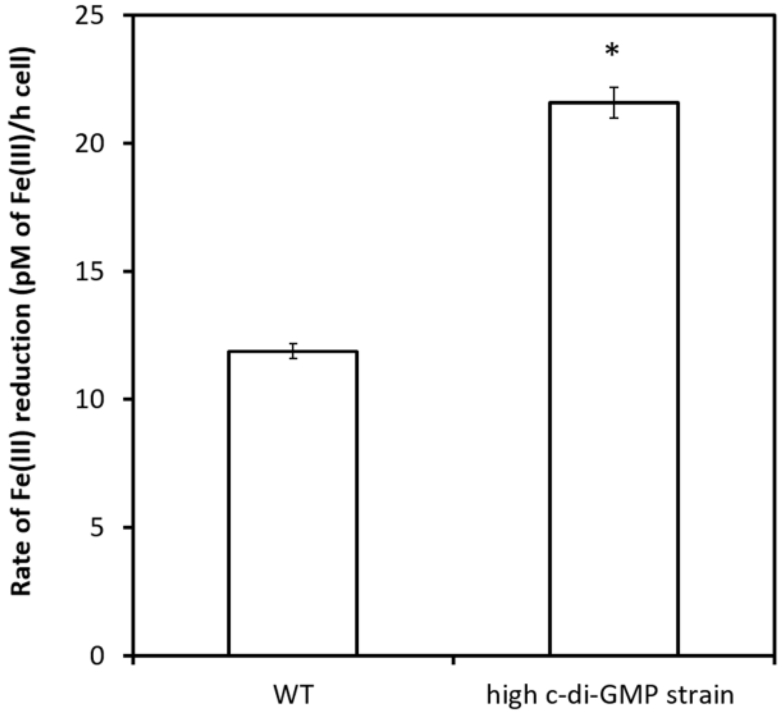

Figure S3. Rate of Fe(III) reduction by *S. oneidensis* MR-1 WT and high c-di-GMP strain MR-1/pYedQ$_2$. * indicates statistical significance (t-test p-value<0.05, n=3).

References

Chen, Y., Yuan, M., Mohanty, A., Yam, J.K.H., Liu, Y., Chua, S.L., et al. (2015) Multiple diguanylate cyclase-coordinated regulation of pyoverdine synthesis in Pseudomonas aeruginosa, *Environ. Microbiol. Rep.* **7**: 498-507.

Heidelberg, J.F., Paulsen, I.T., Nelson, K.E., Gaidos, E.J., Nelson, W.C., Read, T.D., et al. (2002) Genome sequence of the dissimilatory metal ion–reducing bacterium Shewanella oneidensis, *Nature biotechnology* **20**: 1118-1123.

Mortazavi, A., Williams, B.A., McCue, K., Schaeffer, L., and Wold, B. (2008) Mapping and quantifying mammalian transcriptomes by RNA-Seq, *Nat. Methods* **5**: 621-628.
